# Amplification of mtDNA control region in opportunistically collected bird samples belonging to nine families of the order Passeriformes

**DOI:** 10.1080/23802359.2017.1289342

**Published:** 2017-02-16

**Authors:** Ashutosh Singh, Ajit Kumar, Ramani Suresh Kumar, Dinesh Bhatt, Sandeep Kumar Gupta

**Affiliations:** a Department of Animal Ecology and Conservation Biology, Wildlife Institute of India, Dehradun, India;; b Department of Environmental Science and Zoology, Gurukula Kangri University, Haridwar, India;; c Department of Endangered Species Management, Wildlife Institute of India, Dehradun, India

**Keywords:** Mitochondrial control region, birds, non-invasive samples, PCR

## Abstract

We describe six sets of primers for amplifying the mitochondrial control region (CR) of various bird species belonging to nine families of the order Passeriformes. These overlapping primers, with both short and long fragments, yielded an approximately 1 kb fragment of the CR. The short length of the amplified product makes the primers suitable for degraded DNA samples. These primers were used on a wide range of bird species for amplifying and sequencing the highly variable portion of the CR. The primers proved to be a valuable tool for studying the population genetics of bird species. The different sets of primers provide the researcher a choice of markers for different sample types and studies.

## Introduction

Molecular genetics is being used increasingly in various conservation applications. It is useful in determining the level of genetic variation, phylogenetics and phylogeography with a high level of accuracy. Due to the presence of conserved sites in mitochondrial DNA (mtDNA) regions such as the 12s, 16s and cytochrome *b* gene, these fragments are widely used for species identification (Kocher et al. 1989; Wan et al. 2004). The mtDNA control region is helpful in identifying significant conservation units for those species that are historically isolated (Wenink et al. 1994; Gupta et al. 2015). Besides, knowledge about the variability of the hypervariable control region is helpful in identifying lower-category taxa such as species or sub-species. The mitochondrial genome is highly variable in avian species (Wenink et al. 1994). The control region (CR) often evolves faster than the rest of the mitochondrial genome (Baker & Marshall 1997). This variability of the CR has made it a powerful tool for studying the genetic structures of populations. Because of the high variability in the CR and the least conserved sites, designing conserved primers is often a challenging task (Arif & Khan 2009). Moreover, bird genetics largely involves non-invasively collected biological samples (shed feathers, faeces, shells of broken/hatched eggs). Amplifying long fragments of the CR from such opportunistically collected samples is a challenging task. A set of primers amplifying shorter fragments will be useful for such samples (Gupta et al. 2014). In this work, we describe a panel of primers for amplification of the mtDNA CR of selected bird species from degraded DNA.

## Materials and methods

### Primer design

The complete mitochondrial genomes of 28 bird species belonging to nine families (Muscapidae, Polioptilidae, Emberizidae, Estrildidae, Viduidae, Nectariniidae, Passeridae, Prunellidae, Fringillidae) were obtained from GenBank and aligned using Clustal W multiple alignments (Thompson et al. 1994). On the basis of sequence similarity, we designed four forward and four reverse primers targeting the CR (Table 1).

**Table 1.** Primer sequences for the amplification of control region of bird species and expected length of their amplicons.

S. No.	Primer name/combination	Sequence (5′-3′)/Expected amplicon length
1	FLCF1	GAA TGG GGT CAA AGT GCA TCA GT
2	FLCF2	TGA TGG ACA TGT CAA GAG GAA G
3	FLCF3	GG CGC AAA AGA GCA AGT
4	FLCR1	ACT TGC TCT TTT GCG CC
5	FLCF4	GTA GCT CGG TTC TCG TGA GAA
6	FLCR2	TTC TCA CGA GAA CCG AGC TAC
8	FLCR3	CCT GAA AAG CCG CTG TTA T
9	FLCR4	TCC ATC TCC AGC TCC CAA AGC
*Primer combination and expected length*		
i.	FLCF1+ FLCR1	320 (bp)
ii.	FLCF1 + FLCR2	410 (bp)
iii.	FLCF2+ FLCR1	217 (bp)
iv.	FLCF2+ FLCR2	300 (bp)
v.	FLCF4+ FLCR3	510 (bp)
vi.	FLCF3+ FLCR4	569 (bp)

### Sample collection, DNA extraction and amplification

We collected shed feathers, broken egg shells and blood samples of 28 different bird species through a field survey. Genomic DNA was extracted using the standard phenol–chloroform method (Sambrook et al. 1989) and subjected to PCR amplification using the primer combination described in Table 1. The amplification was carried out in a 20 μl reaction volume containing 1 μl of the extracted DNA, 100 μM of dNTPs, 4 pmol of each primer, 1.5 mM MgCl_2_, 0.5 units of AmpliTaq Gold (Life Technologies) and 1 × PCR buffer (10 mM Tris–HCl, pH 8.3, and 50 mM KCl). The PCR conditions were the following: initial denaturation at 95 °C for 10 min, followed by 35 cycles of denaturation at 95 °C for 45 s, annealing at 56 °C for 45 s and extension at 72 °C for 90 s. The final extension was at 72 °C for 10 min. The PCR products were electrophoresed on 2% agarose gel, stained with ethidium bromide (0.5 mg/ml) and visualized under a UV transilluminator. The PCR products obtained were sequenced directly in 3130 Genetic Analyzer (Applied Biosystems) from both directions.

## Results and conclusion

**Figure 1.**
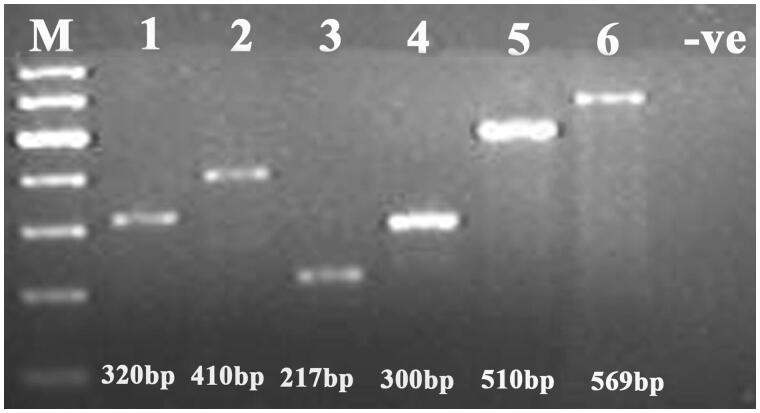
Gel image showing the amplification result of mtDNA control region from the DNA extracted from bird samples using primer FLCF1 + FLCR1 (lane 1); FLCF1 + FLCR2 (lane 2); FLCF2 + FLCR1 (lane 3); FLCF2 + FLCR2 (lane 4); FLCF4 + FLCR3 (lane 5); FLCF3 + FLCR4 (lane 6).

DNA from 28 different bird species was successfully amplified. The primers described in this study were useful in generating the DNA sequence database and were helpful in identifying species and sub-species and in phylogeographic differentiation. The use of different lengths of the CR amplicon in single PCR was a useful approach to amplifying the combination of short and longer DNA fragments found in degraded samples (Figure 1). The short length of the amplified product makes these primers suitable for highly degraded samples. Therefore, the overlapping fragments generated by the primer set were helpful in covering the longer portions of the CR. The PCR conditions described in this article worked consistently for all the primers mentioned. Besides, these primers could be used with a large range of bird species. Hence, they are can be a valuable tool for studying population genetics and identifying evolutionarily significant units (ESUs).
